# Corrigendum to “Cytotoxicity Assessment of PM_2.5_ Collected from Specific Anthropogenic Activities in Taiwan”

**DOI:** 10.3390/ijerph17144932

**Published:** 2020-07-08

**Authors:** Tuan Hung Ngo, Pei Chun Tsai, Yune-Fang Ueng, Kai Hsien Chi

**Affiliations:** 1 Institute of Environmental and Occupational Health Sciences, National Yang Ming University, Taipei 112, Taiwan; tuanhung0712@gmail.com (T.H.N.); poipeiun@gmail.com (P.C.T.); 2 International Health Program, National Yang Ming University, Taipei 112, Taiwan; 3 Divisions of Basic Chinese Medicine, National Research Institute of Chinese Medicine, Taipei 112, Taiwan; ueng@nricm.edu.tw; 4 Institute of Medical Sciences, Taipei Medical University, Taipei 112, Taiwan

In the Section 2.4.3 part, we described the method of the *umu* assay as following:

“Genotoxicity effects of PM_2.5_ organic extracts were determined using *umu* assay. *Umu* assay was performed on Salmonella typhimurium strain TA1535 containing NM2009 plasmids. In the first step, bacteria were grown overnight in LB medium containing ampicillin (50 µg/mL). The overnight cultures were then diluted 50 folded with TGA medium (tryptone 1%, glucose 0.2%, NaCl 0.5%) containing ampicillin. The bacteria were cultured until the absorbance at 600 nm reached about 0.21. After that, the β-galactosidase activity expressed by genotoxic activation was measured using 2-nitrophenyl-β-d-galactopyranoside as a target substance, and 2-nitrophenyl-β-d-galactopyranoside as a bacterial growth condition. The expression of the β-alactosidase enzyme was measured by the color of the receptor when it was excited. The light absorbance at wavelength A420 nm was measured. The genotoxicity was examined by the induction ratio (IR) between β-galactosidase activity of sample group and that of control group. IR > 1.5 is considered as presence of genotoxicity effect [25,26].”

After publication, Dr. Yoshimitsu Oda, author of the *umu* test, has pointed out some imprecise method description. Therefore, we made the following correction:

“The genotoxicities of PM_2.5_ organic extracts were determined by using the *umu* assay. The *umu* assay was proceeded using the *Salmonella typhimurium* strain TA1535/pSK1002 [1]. In the first step, bacteria were grown overnight in LB medium containing ampicillin (50 μg/mL). The overnight cultures were then diluted 50-fold with TGA medium (1% Tryptone, 0.2% glucose, 0.5% NaCl) containing ampicillin. The bacteria were cultured until the absorbance at 600 nm (for monitoring the bacterial growth) reached about 0.21. After that, the β-galactosidase activity expressed by genotoxic activation was measured using 2-nitrophenyl-β-d-galactopyranoside as a substrate, and the absorbance at 420 nm was determined. The induction of *umu* gene expression was expressed as the β-galactosidase activity normalized by bacterial growth [1].”

We also updated the forth part of supplementary method about the *umu* test as follows:

“The *umu* test was used for genotoxicity testing in this study [2]. The genotoxicities of the extracts of fine particulates were tested using *Salmonella typhimurium* TA1535/pSK1002 in the presence of a recombinant cytochrome P450 (CYP) 1A1 monooxygenase system. Different extracts of fine particulates were dissolved in DMSO or a suitable solvent to make a solution, and if necessary, the insoluble matter was filtered. The solution of extract was added to the *umu* assay mixture, and the mixture was cultured at 37 °C for 2 h. The blank control assay was carried out by exposing the bacteria to the solvent. The genotoxicity in the presence or absence of the recombinant CYP 1A1 monooxygenase system was compared. In cases where DMSO was used as the solvent to prepare the extract solution, the final concentration of DMSO was less than 1% (*v*/*v*). To consider the potential effects of the extracts on bacterial growth, the absorbance at 600 nm (A600 nm) of the bacterial reaction mixture was measured. The β-galactosidase activity expressed by genotoxic activation was measured using 2-nitrophenyl-β-d-galactopyranoside as the substrate and the β-galactosidase activity was monitored by measuring the absorbance at a wavelength of 420 nm at the end of assay. The induction of the *umu* gene expression was expressed as the β-galactosidase activity standardized by the bacterial growth. When the test substance elevated the standardized activity level by greater than 1.5-fold of that of the blank control assay, it was considered to be genotoxic.”
