# Supplementary material for: Cytotoxicity Assessment of PM2.5 Collected from Specific Anthropogenic Activities in Taiwan
Source: Int J Environ Res Public Health. 2019 Dec 11;16(24):5043. doi: 10.3390/ijerph16245043 (PMC6950068; doi:10.3390/ijerph16245043)
Supplement: Supplementary file 1 [file ijerph-16-05043-s001.pdf]

CYTOTOXICITY ASSESSMENT OF $PM_{2.5}$ COLLECTED FROM SPECIFIC ANTHROPOGENIC ACTIVITIES IN NORTH TAIWAN

Tuan Hung Ngo [1 2], Pei Chun Tsai [1], Yune-Fang Ueng [3], Kai Hsien Chi [1*]

[1] Institute of Environmental and Occupational Health Sciences, National Yang Ming University, Taipei, 112, Taiwan.

[2] International Health Program, National Yang Ming University, Taipei, 112, Taiwan.

[3] Divisions of Basic Chinese Medicine, National Research Institute of Chinese Medicine, Taipei, 112, Taiwan.

SUPPLEMENTARY DOCUMENTS

Table S1: Share of each filter for analysis and research

<table>
<tr><th></th><th>Chemical analysis</th><th>Cytotoxicity experiment</th><th>Total</th></tr>
<tr><td>Ion</td><td>1/16</td><td>1/16</td><td rowspan="4">16/16</td></tr>
<tr><td>Metal</td><td>1/16</td><td>2/16</td></tr>
<tr><td>Organic compounds</td><td>6/16</td><td>2/16</td></tr>
<tr><td>Back up</td><td colspan="2">3/16</td></tr>
</table>

Description of different cell test

1. Cell culture

A549 cell line was cultured in a 10 cm cell culture dish (dish), using Ham's F12 medium and adding 10% Fetal Bovine Serum (FBS) and 1% Penicillin / Streptomycin (P / S) An antibiotic is cultured in an environment of 37 ° C and 5% CO2 controlled humidity, and the cells are subcultured every 2 to 3 days and the medium is changed. The cells must be washed twice with PBS to remove the serum components in the culture medium for trypsin action. After rinsing the cells with PBS, completely cover the cells attached to the bottom of the culture plate with 1 mL of 0.05% trypsin / EDTA (trypsin / EDTA). After 5 minutes of treatment, tap the culture plate to remove the cells from the bottom of the plate. Trypsin / EDTA was re-dissolved in a 1: 1 medium to stop the enzyme reaction, and then placed in a centrifuge at 12000 rpm for 3 minutes to remove the trypsin-containing medium. After resuspending in 10 mL of medium, take an appropriate amount. The number of cells is cultured in a new culture plate. When the cells are attached for about 8 minutes, they need to be passaged again. Before the experiment of the same batch of cells, the doubling time is tested to determine whether the cell growth rate meets the ATCC recommendations. time: about 22 hours) to initially confirm the health status of the batch of cells.

2. Cell viability analysis

MTT assay for Cell viability analysis is a widely used method for determining cell viability. MTT (methylthiazole tetrazolium bromide) is a yellow compound, which can be reduced to fat-soluble purple formazan crystals by using succinate dehydrogenase (SDH) and cytochrome C in the carcass of living cells. However, there is no glandular response in dead cells, so the formation of formazan crystals is directly proportional to the order of live cells, and then the absorbance value was measured by ELISA Reader to determine the difference between the treated group and the control group.

Test operation steps:

1. Seed 8 × 103 cells with 100 µL of medium in each 96-well culture dish and culture for 24 hours.

2. In order to obtain the dose-response curve of sample toxicity, dilute each sample to 20%, 40%, 60%, 80% with the applicable solvent, and 100% concentration, and the

DMSO control group and the group without additional treatment 1 ‰ dilution and exposure to A549 cells attached to the culture plate

3. After 24 hours of exposure, remove the extract and add 10% diluted MTT (5 mg / mL-PBS) in complete medium and incubate for 40 minutes in the dark

4. After removing the MTT, add 100 µL / well of DMSO to dissolve the purple crystals.

5. Use ELISA Reader to measure the absorbance (set wavelength is 570-650 nm)

3. Cell Oxidative Stress Test

Excessive release of free radicals by cells under stimulation is a key factor in the generation of oxidative stress. In order to determine the amount of free radicals generated after the cells are exposed to fine suspended particulate extracts, that is, their potential oxidative stress, they will be cultured in complete medium (F12 medium containing 10 % FBS and 1% P / S) A549 cells were exposed to PM2.5 extract for 6 hours. After removing the exposure solution, the cells were exposed to 5 µM 2 ', 7'-dichlorodihydrofluorescein diacetate (DCFDA) superoxide. Anion determination of fluorescent dyes, cultured for 30 minutes in the dark, oxidized and metabolized DCFDA to DCF (2,7-dichlorofluorescin) with fluorescence, and the intensity of the fluorescence could reflect reactive oxygen species (ROS) When the content is high or low, the fluorescence intensity of cells exposed to PBS solution is measured by flow cytometry, and the oxidative stress index ROS level (BD, FACSVerse system, CA) is compared with the control group under the exposure of pollutants. , USA).

Test operation steps:

1. Expose the sample to 5 × 105 cells / 6 well-plate, while exposing the positive control and blank sample control

2. After 6 hours, remove the exposure solution and wash it once with PBS.

3. Expose at 0.05% Trypsin / EDTA for 5 minutes to transfer cells into a 15 mL centrifuge tube

4. After the cells are transferred into the centrifuge tube, rinse with PBS twice to remove the serum components present on the cells.

5. After exposure to DCFDA for 30 minutes in the dark, rinse twice with PBS

6. The fluorescence value (485nm-530nm) was measured by flow cytometry on the machine.

4. Umu test

Umu test was used for genotoxicity testing in this study (Shimada et al., 1994). The genotoxicity of the fine suspension particulate extracts will be tested using the S. typhimurium TA 1535 / pSK1002 and NM2009 strains in the presence of the recombinant cytochrome P450 CYP1A1 monooxygenase system.

Dissolve in different fine suspended particulate extracts using DMSO or a suitable solvent to make a solution, and if necessary, filter insoluble matter. The solution was added to the activated strain, and the medium was cultured at 37 ° C, and the strain was exposed to the fine suspension particulate extraction solution for 2 hours. The blank control group was prepared by simply exposing the solvent to the strain. The genotoxicity in the presence or absence of the recombinant cytochrome CYP 1A1 monooxygenase system was compared. If DMSO is used as the solvent to prepare the exposure solution, the final concentration of DMSO needs to be less than 1% (v / v). In order to consider the possibility that the test object affects the growth of bacteria, the reaction bacterial solution is measured at an absorbance of 600 nm (A600 nm). The β-galactosidase activity expressed by genotoxic activation was measured using 2-nitrophenyl-β-d-galactopyranoside as the target substance, and umu gene proliferation was used as the bacterial growth situation to standardize. As a result of the measurement, the expression of the β-galactosidase enzyme is determined by the coloration of the receptor when it is excited, and the absorbance (A420nm) is measured at a wavelength of 420nm after the end of the test step. When the test substance caused β-galactosidase expression activity to be more than 1.5 times higher than that of the blank control group, it was judged to be genotoxic.

Table S2: Cell viability of mtt assay

	PM2.5 mass (ng)	Log PM2.5	Effect of ion	Effect of metal	Effect of organic compounds
LRT	19.49	1.29	109.39±12.13		
	38.98	1.59	109.20±12.07		
	58.47	1.77	107.38±13.88		
	77.96	1.89	102.95±8.11		
	97.46	1.99	101.65±10.22		
	61.84	1.79			98.23 ± 5.19
	123.69	2.09			100.02 ± 3.77
	185.53	2.27			98.55 ± 5.93
	247.37	2.39			96.51 ± 6.90
	309.21	2.49			91.06±9.45
	2.77	0.44		92.94±3.29	
	5.54	0.74		91.86±4.26	
	8.30	0.92		92.86±3.3	
	11.07	1.04		93.7±3.4	
	13.84	1.14		92.06±3.56	
Traffic	45.22	1.66	103.49±13.2		
	90.43	1.96	98.88±11.35		
	135.65	2.13	98.83±10.19		
	180.87	2.26	96.89±11.42		
	226.08	2.35	103.91±14.71		
	80.51	1.91			102.27±2.82
	161.02	2.21			100.91±3.85
	241.54	2.38			101.41±4.69
	322.05	2.51			98.96±5.97
	402.56	2.60			100.64±2.87
	2.94	0.47		95.86±3.59	
	5.88	0.77		95.52±2.4	
	8.82	0.95		94.31±5.89	
	11.76	1.07		94.05±3.6	
	14.70	1.17		93.05±2.48	
Night	3.58	0.55	100.55±11.46		

Market	7.15	0.85	95.51±6.98		
	10.73	1.03	99.11±13.75		
	14.31	1.16	99.6±10.2		
	17.88	1.25	94.07±10.45		
	63.67	1.80			97±2.25
	127.35	2.10			96.89±3.85
	191.02	2.28			95.21±3.56
	254.70	2.41			94.94±3.92
	318.37	2.50			91.27±3.18
	0.26	-0.58		96.23±3.24	
	0.52	-0.28		99.16±5.24	
	0.79	-0.11		97.47±5.67	
	1.05	0.02		95.19±4.36	
	1.31	0.12		92.48±2.8	

Table S3: Concentration of $PM_{2.5}$ and organic compounds

	LRT	Traffic	Night market
$PM_{2.5}$ (μg/m^3)	39.0	43.0	26.3
Total PCDD/Fs concentration (fg I-TEQ/m^3)	7.57	46.9	10.1
Total PCDD/Fs content in $PM_{2.5}$ (fg I-TEQ/m^3)			
Total PAHs (BAPeq ng/m^3)	<0.1	<1.0	3.57

Table S4: Ion concentration in $PM_{2.5}$ samples

**μg/m^3**	**LRT**	**Traffic**	**Night market**
	**(n=2)**	**(n=2)**	**(n=2)**
**Na^+**	0.178	0.091	0.306
**NH_4^+**	2.11	0.680	1.43
**K^+**	0.208	0.056	0.116
**Mg^{2+}**	0.053	0.020	0.022
**Ca^{2+}**	0.087	0.157	0.034
**Cl^-**	0.306	0.124	0.147
**NO_3^-**	1.93	0.787	0.533
**PO_4^{3-}**	0.017	0.017	0.041
**SO_4^{2-}**	5.26	1.51	4.98
**Total**	10.2	3.44	7.61

Table S5: Concentration of metal elements in three $PM_{2.5}$ samples

ng/m^3	LRT	Traffic	Night market
**Al**	157	749	19.6
**Fe**	200	693	13.9
**Na**	897	379	50.0
**Mg**	95.0	248	6.54
**K**	464	338	25.0
**Ca**	134	792	22.4
**Sr**	1.85	4.32	0.198
**Ba**	3.06	19.6	0.441
**Ti**	10.6	62.0	0.639
**Mn**	17.8	17.3	0.603
**Co**	0.296	0.387	0.009
**Ni**	7.09	4.90	0.193
**Cu**	16.8	16.6	0.589
**Zn**	99.4	72.4	3.52
**Mo**	24.6	71.1	0.047
**Cd**	0.938	0.395	0.031
**Sn**	3.65	3.13	0.105
**Sb**	3.04	2.60	0.104
**Tl**	0.295	0.115	0.011
**Pb**	40.4	16.2	0.822
**V**	11.1	9.99	0.261
**Cr**	4.38	5.41	0.568
**As**	5.54	1.34	0.138
**Y**	0.498	0.266	0.002
**Se**	1.58	0.655	0.103
**Zr**	4.54	3.40	0.053
**Ge**	0.906	2.40	0.012
**Rb**	1.60	1.66	0.070
**Cs**	0.145	0.126	0.006
**Ga**	1.01	1.07	0.048
**La**	0.253	0.636	0.029
**Ce**	0.275	1.11	0.044
**Nd**	0.175	0.448	0.001
**Total (μg/m^3)**	2.21	3.52	0.146

Table S6: Taiwan general public exposure parameter

	**Children**		**Adults**	
	Male	Female	Male	Female
**Age (years)**	6-14		15-64	
**Body Weight (kg)**	35.8	33.5	68.6	56.8
**Active Inhalation Rates (m^3/day)**	16.8	14.7	18.3	12.3

Detection limit of organic compound analysis

Dioxin detection limit

Variability in the duplicate analysis of PCDD/F concentrations was all lower than 10% (1.56-8.9%). For quality control, a laboratory blank and matrix spike sample (2.0~20 pg/μl PCDD/Fs) were used in the analytical procedure for every eight samples for quality control. Method detection limits (0.03~1.26 pg/g) were determined from the blanks and quantified as three times the standard deviation of the concentration in the blanks. In this study, the concentrations of all laboratory blank samples are less than 1.0 pg (PCDD/Fs). The mean recoveries of standards for all 13C12-2,3,7,8-substituted PCDD/Fs range from 53% to 107%. The analyzed results were all within the acceptable 40%-130% range, established by the US EPA in Methods 23.

PCDD/F detection limitation is listed as below.

2,3,7,8-TeCDD	0.093629
1,2,3,7,8-PeCDD	0.074502
1,2,3,4,7,8-HxCDD	0.013906
1,2,3,6,7,8-HxCDD	0.011841
1,2,3,7,8,9-HxCDD	0.013174
1,2,3,4,6,7,8-HpCDD	0.001455
OCDD	0.000387
2,3,7,8-TeCDF	0.016327
1,2,3,7,8-PeCDF	0.004470

2,3,4,7,8-PeCDF	0.044063
1,2,3,4,7,8-HxCDF	0.008277
1,2,3,6,7,8-HxCDF	0.006882
2,3,4,6,7,8-HxCDF	0.008370
1,2,3,7,8,9-HxCDF	0.012232
1,2,3,4,6,7,8-HpCDF	0.000554
1,2,3,4,7,8,9-HpCDF	0.000918
OCDF	0.000249

PAH detection limit

Nap	0.093216
AcPy	0.015634
AcP	0.011204
Flu	0.125498
Phe	0.05211
Ant	0.060264
Flt	0.02963
Pyr	0.02762
BaA	0.01225
Chr	0.012796
BbF	0.03027
BkF	0.033208
BaP	0.047674
IND	0.083984
DBA	0.049562
BghiP	0.0913
